# Benefit of Wearing an Activity Tracker in Sarcoidosis

**DOI:** 10.3390/jpm10030097

**Published:** 2020-08-22

**Authors:** Marjolein Drent, Marjon Elfferich, Ellen Breedveld, Jolanda De Vries, Bert Strookappe

**Affiliations:** 1 ILD Care Foundation Research Team, 6711 NR Ede, The Netherlands; elfferichm@zgv.nl (M.E.); breedvelde@zgv.nl (E.B.); j.devries@tilburguniversity.edu (J.D.V.); strookappeb@zgv.nl (B.S.); 2 ILD Center of Excellence, Department of Pulmonology, St. Antonius Hospital, 3435 CM Nieuwegein, The Netherlands; 3 Department of Pharmacology and Toxicology, FHML, University Maastricht, 6211 LK Maastricht, The Netherlands; 4 Department of Physical Therapy, Hospital Gelderse Vallei, 6716 RP Ede, The Netherlands; 5 Department of Medical Psychology, Elisabeth-TweeSteden Hospital Tilburg, 5042 AD Tilburg, The Netherlands; 6 Department of Medical and Clinical Psychology, Tilburg University, 5037 AB Tilburg, The Netherlands

**Keywords:** activity tracker, exercise capacity, fatigue, physical activity, physical training, sarcoidosis

## Abstract

Sarcoidosis causes many disabling symptoms, including fatigue and exercise limitations, which have been shown to improve by physical activity programs. The aim of this study was to estimate the effect of continuous activity monitoring using an electronic activity tracker (AT) on exercise performance and fatigue of sarcoidosis patients, compared to controls (cohort study), and the effect of additional personal coaching (randomized trial) over a period of 3 months. Fifty-four sarcoidosis patients received an AT (Group Ia: 27 with coaching and Group Ib: 27 without). A historical group of sarcoidosis patients (Group II; *n* = 41) who did not follow a physical activity program served as controls. Exercise performance of patients wearing an AT (Group I) improved compared with controls (Group II), including the 6MWD, % predicted (∆4.4 ± 9.1 versus ∆0.7 ± 5.0, respectively), and fatigue levels decreased (∆−3.9 ± 5.7 versus ∆−1.8 ± 5.3). Patients with coaching (Group Ia) showed greater improvement of exercise capacity over time than patients without coaching (Group Ib) as shown by the Steep Ramp Test results (watts: ∆20.2 ± 33.8 versus ∆5.7 ± 26.4; and SRT, VO_2_max, % predicted: ∆1.6 ± 2.6 versus ∆0.7 ± 2.3). Sarcoidosis patients wearing an AT achieved improvement of exercise performance and reduction of fatigue. We therefore recommend encouraging sarcoidosis patients to wear an AT to stimulate physical activity and reduce fatigue. The additional benefit of coaching needs to be explored in future studies.

## 1. Introduction

Sarcoidosis patients often suffer not only from organ-related symptoms but also from disabling non-specific, non-organ-related symptoms, such as fatigue, reduced muscle strength, loss of physical condition, reduction of physical activity (PA) in daily life, and pain [1,2,3,4,5,6,7,8]. Fatigue is the most frequently reported symptom in sarcoidosis patients, regardless of the clinical presentation, varying from 50 to 90% [3,5,9]. This may persist after other signs of sarcoidosis activity have resolved and adversely impacts major life areas, including quality of life (QoL) and work ability [2,4,10,11,12,13,14].

So far, there is promising evidence for the benefits of physical training in sarcoidosis [15,16,17,18]. Interventions involving technology that is readily accessible on a daily basis to monitor activity levels can support care providers in encouraging patients to achieve behavioral changes [19]. These interventions may be an effective strategy to provide PA coaching without increasing time demands on primary care providers [20,21]. Moreover, they give patients an opportunity to keep up a more active lifestyle with direct feedback and monitor their physical performance over time. Counseling, guidance and support using e-health technology has been found to be very helpful for patients who want to improve their PA [22,23]. The use of commercially available, technology-based wearable activity trackers (ATs) is growing, for research and recreational purposes, both among healthy persons and among those with chronic illness. Beneficial effects of AT-based counseling have been demonstrated in patients with chronic diseases [24,25]. In a recent study by our group, we found that wearing an AT stimulated patients to be more physically active [26].

The aim of this study was to estimate (1) the potential effect of continuous activity monitoring using an electronic AT, compared with a historical control group in a cohort study and (2) the effect of additional personal coaching of sarcoidosis patients in a randomized trial. The outcome measures were exercise performance and fatigue.

## 2. Materials and Methods

### 2.1. Study Design, Subjects and Controls

Patients were recruited from the ILD Center of Excellence of the St. Antonius Hospital Nieuwegein, the Netherlands, from January 2017 until March 2019. The diagnosis of sarcoidosis was confirmed by a multidisciplinary team in accordance with accepted guidelines [27].

Patients were eligible to participate if they (1) were in a clinically stable condition without changes in complaints or changes in initiated medical management during the preceding three months and (2) if they were between 18 and 75 years old, had sufficient command of the Dutch language, and had internet access at home or a compatible smartphone/tablet. Informed consent was obtained from all participants. Patients who had participated in a training program during the 6 months prior to inclusion were excluded. Treatment allocation was done by one of the authors (EB), using minimization as randomization method. Male and female patients were allocated to the groups in equal proportions (Ia and Ib) to ensure equal distribution. Clinical trial ID: NCT04475653.

A historical control group of sarcoidosis patients included in an earlier retrospective observational study by Strookappe et al. (*n* = 41; evaluated by the ild care expertise team of Gelderse Vallei Hospital, the Netherlands), who did not follow a physical training program, was used as a control group in the present study (Group II) [17].

Our cohort study compared the participating patients (Group I) with the historical control group of sarcoidosis patients (Group II). In addition, a prospective randomized clinical trial (clinical trial ID: NCT04475653) was performed within Group I, comparing Group Ia (intervention by coaching) with Group Ib (without coaching), to estimate the effect of adding coaching to continuous activity monitoring with an electronic AT. In both parts of the study, the outcome measures were exercise performance and fatigue. Data were collected in both groups at baseline and after 12 weeks.

### 2.2. Lung Function Tests

Forced vital capacity (FVC) and forced expiratory volume in one second (FEV_1_) were measured with a pneumotachograph. The diffusing capacity of the lung for carbon monoxide (DLCO) was measured using the single-breath method (Masterlab, Jaeger, Würzburg, Germany). Values were expressed as a percentage of the predicted value (i.e., FVC%, FEV_1_%, and DLCO%, respectively).

### 2.3. Exercise Capacity

Maximal oxygen uptake, and other commonly collected variables, were measured during a cardiopulmonary exercise test using the Steep Ramp Test (SRT) protocol on a cycle ergometer, as reported earlier [17]. The 6-minute walk test (6MWT) was administered according to the American Thoracic Society Guidelines [28]. Predicted 6MWD values were calculated according to Gibbons and colleagues [29]. To avoid gender as well as age bias, the outcome measures of physical performance were also expressed as percentage of predicted. This equation took age and sex into account.

### 2.4. Outcome Measures: Activity and Fatigue Monitoring

Patients’ daily activities were measured by an electronic AT—Fitbit Charge HR by Fitbit Inc. (San Francisco, CA, USA). The Fitbit is a commercial tool, used to track physical activity, providing personalized data on step count, activity intensity and activity duration. The Fitbit Charge HR appeared to have good test-retest reliability for step count regarding slow, moderate and more vigorous walking speeds [30]. We acknowledge that activity trackers are not medical devices and systematic errors have been reported [31]. In our view, these limitations were acceptable with regard to the purpose of this study. Number of steps, estimated distance, number of flights climbed, and activity minutes were visible for patients on their smartphone (IOS and Android app [Mansystems Nederland B.V., Barneveld, The Netherlands]) website. Patients had access to their daily activity results and activity history. Their physical therapist was able to monitor these parameters on a web-based client. Fatigue was measured with the 10-item Fatigue Assessment Scale (FAS) [9]. This questionnaire was filled out on a weekly basis. The FAS has acceptable psychometric properties in sarcoidosis [32]. Moreover, the FAS has shown good reliability and validity in a sarcoidosis population [33].

### 2.5. Intervention

All participants received a Fitbit for free. They were encouraged to improve their physical fitness, which was assessed at baseline. The patients in the intervention group (group Ia) engaged in a 3-month physical therapist-guided activity program. The guidance was based on the input from the AT and the questionnaires. The weekly FAS scores, Fitbit-measured parameters and brief daily questions were combined in a web-based dashboard accessible to the assigned physical therapist. The information was used for goal setting, encouraging, and identifying barriers and facilitators for patients to become more active. The coaching procedure included weekly action planning and feedback, modeling of behaviors and problem solving, and individual decision making, by email and/or telephone. The coaching physical therapist acted as facilitator, and assisted participants in making choices and achieving success in terms of reaching self-selected goals.

Although the patients in group Ib did not receive the guidance from a physical therapist, their daily activity was also computed with the Fitbit Charge HR, and patients were also able to use the Fitbit and a monitoring app to monitor their performance, questionnaire results and progress.

Self-report feedback questionnaires at follow-up (12 weeks) were used to determine participants’ experience with data from the Fitbit tracker itself and coaching, if applicable.

### 2.6. Statistical Analysis

Descriptive statistics were used for baseline characteristics. Differences between the two patient groups (Groups Ia and Ib), and between Group I and the controls (Group II), were examined using independent samples *t*-tests (Mann-Whitney U tests) or Chi-square (or Fisher’s exact) tests on the baseline data, depending on the type of variable and the normality of the data. Analyses of variance for repeated measures were performed between all groups to examine the effect of the training over time on fatigue (FAS), submaximal exercise capacity (6MWD), and maximal exercise capacity (SRT). The interaction with time would indicate whether both groups of patients differ or not concerning their scores across time. In our study, we first examined whether there was a difference between the two groups at baseline. Since this was not the case, no adjustment was deemed necessary. A difference between the two groups at follow-up indicates a difference in time between the two groups. This difference between the groups at follow-up is a difference that occurred in time.

To avoid gender as well as age bias, the outcome measures of physical performance were also expressed as percentage of predicted. This equation took age and sex into account.All statistical analyses were performed using SPSS statistical software (version 24.0 for Windows) (SPSS Inc., Chicago, IL, USA).

## 3. Results

Sixty-three patients were enrolled in this study. Nine patients were excluded because they did not have access to an appropriate smartphone. Finally, 54 patients were randomized by one of the authors (EB) to group Ia or group Ib in such a way as to ensure equal distribution of men and women in both groups. Demographic and clinical baseline characteristics of the 54 included patients (Group I), the nine excluded patients, and the control patients (Group II; *n* = 41; without monitoring or training) are summarized in Table 1. None of the patients had cardiac involvement or any form of neurosarcoidosis. At baseline, the included patient groups did not differ from the control group regarding demographic and clinical variables, except for sex (*p* = 0.01 see also Table 1). No differences were demonstrated between Groups Ia, Ib and II with regard to fatigue and exercise capacity. Fatigue scores (FAS) did not correlate with the demographic and clinical variables of age, BMI, time since diagnosis, inflammatory markers, and lung function tests. Exercise capacity (6MWD, SRT) did not correlate with lung function tests, time since diagnosis, or baseline fatigue levels. Sex, age and BMI were not significantly related to exercise performance; and baseline 6MWD, % of predicted, explained only 13% of the change in exercise performance, F = 4.984, *p* = 0.011.

### 3.1. Comparing Activity Tracker Users (Group I) and Controls (Group II)

In contrast to the controls (Group II), the walking distance—both 6MWD distance and 6MWD, % of predicted—as well as the SRT and VO_2_max (mL/kg/min) increased significantly in the study sample wearing ATs (Group I), whereas fatigue decreased (*p* < 0.01 for all; see Table 2). However, an interaction effect for time with group was found for both 6MWD (F = 4.154, *p* = 0.044) and 6MWD, % of predicted (F = 5.016, *p* = 0.028). No interaction effect for time with group was found for fatigue (FAS, F = 3.743, *p* = 0.056) and for exercise capacity (SRT and SRT VO_2_max mL/kg/min). The SRT (watts) performance in Group I improved between baseline and 3 months, while the SRT (watts) performance in Group II did not change. No interaction effect was found with regard to the SRT (watts, F = 3.014, *p* = 0.089). Similar results were found with regard to SRT VO_2_max (mL/kg/min) (interaction effect: F = 1.145, *p* = 0.290).

The control group (Group II) included more female patients, but analyzing male and female patients separately did not change the results.

### 3.2. Comparing Activity Tracker Users with (Group Ia) and without Coaching (Group Ib)

Patients wearing an AT and receiving personal coaching (Group Ia) showed greater improvement of exercise capacity (SRT, F = 4.515, *p* = 0.039 and SRT VO_2_max mL/kg/min; F = 4.945, *p* = 0.031) over time. Fatigue levels decreased in Group Ia as well as in Group Ib. Exercise capacity, assessed with the 6MWD and 6MWD, % of predicted, increased in both groups. No significant interaction effect was found for the outcome measures 6MWD and FAS (see Table 2).

### 3.3. Evaluation and Feedback Activity Tracker Users

At study completion, responses to feedback questionnaires from patients wearing an AT were evaluated (see also Table 3). Two participants had not completed these. Most patients reported that they found the intervention helpful (47/52:90%), intended to continue tracking their activity (36/52:71%), and would participate in another PA study (42/52:81%). By contrast, most patients were *not* interested in participating in group activities (48/52:92%). They did report that the AT had provided them with greater insight into what sarcoidosis does to them. Forty-nine of the 52 respondents (94%) indicated that exercise worked well for them. Nearly half of the participants from Group 1b (without coaching: 12/26:46%) indicated they liked the feedback from the Fitbit, but would have preferred to get guidance and incentive from a physical therapist.

## 4. Discussion

To the best of our knowledge, this was the first cohort study assessing the effect on exercise performance and fatigue in sarcoidosis of encouraging PA by using a wearable AT. In addition, our prospective randomized trial estimated the effect of additional coaching. The data from the cohort study were compared with those of sarcoidosis patients who did not participate in the AT trial (historical control group). Patients’ symptoms, limitations of daily activities, physical fitness and personal goals were assessed at baseline and after 12 weeks. Half of the patients had contact with a personal physical therapist on a weekly basis and also had the opportunity to contact their coach for questions or advice (two-way contact opportunity; Group Ia). Wearing an AT improved exercise performance and reduced fatigue. It was especially patients who wore an AT and received coaching (Group Ia) who showed improved exercise performance. Our results were in line with previous studies that showed that a supervised physical training program improves fatigue, exercise capacity, and muscle strength [5,8,18].

As reported before, the reason for reduced PA in sarcoidosis is multi-factorial, as there is no clear association with individual factors or sarcoidosis phenotypes [2,4,34]. In line with Cho et al., we found that functional exercise capacity (6MWD) was significantly reduced in sarcoidosis patients and was associated with reduced PA [6]. They assessed PA by a validated triaxial accelerometer and found that daily step counts were significantly lower in participants with sarcoidosis compared to healthy controls. They also found that the 6-MWD had the strongest association with PA in patients with sarcoidosis. ATs can provide unique information about the impact of disease on functioning that is not captured by existing clinical outcome measures for sarcoidosis and can potentially be used to assess response to therapy.

In the present study, patients wearing an AT and receiving coaching improved their exercise capacity (6MWD, 6MWD, % of predicted, and SRT) and had reduced fatigue levels, in contrast to the patients not receiving this coaching. This is in line with Gill et al., who demonstrated that HealtheSteps™ was effective in increasing PA (i.e., step counts per day) and decreasing weekdays sitting time [21]. Moor et al. evaluated patient experiences with a home monitoring program for sarcoidosis and assessed whether home monitoring is a feasible tool to enhance personalized treatment [30]. All patients endorsed the usefulness of an AT, as this stimulated them to be more active and provided good insights into their fatigue. The present study had similar findings. In particular, some of the quotes from the patients participating in their study were comparable with the findings of the present study. For instance, they mentioned that the activity levels corresponded better with their overall functioning than lung function alone [35]. Home monitoring may potentially enable timely recognition of, and response to, changes in symptoms and activity. It is particularly in a heterogeneous disease such as sarcoidosis that home monitoring may pave the way for better individually tailored treatment, enhanced self-management, and improved QoL.

A recent study by our group found that using ATs encouraged patients to be more physically active and gave them a better understanding of their disease, thereby improving their self-management and behavior [26]. However, only 23% of the Dutch sarcoidosis sample we studied indicated to have ever used any kind of AT. We should embrace the use of ATs to meet the challenge of motivating and encouraging sarcoidosis patients to improve their physical activity level. An AT is thus useful to stimulate and improve self-management.

Promotion of exercise and PA, as well as physical therapist-led physical training programs, are important and have many health benefits for patients with chronic illnesses, including sarcoidosis [25,34]. A randomized clinical trial studied the effect of counseling combined with the use of an accelerometer and a smartphone/web-based application on PA in patients with COPD and diabetes mellitus [36]. The group who received the intervention showed greater PA immediately after the intervention than the usual care group, and the effect was still present 3 months after the intervention [36]. Another study about telerehabilitation in patients with pulmonary disease has been announced [37]. In this study, participants will undertake an 8-week group-based pulmonary rehabilitation program, either in-person in a center-based pulmonary rehabilitation program, or remotely from their homes via the internet [37].

### 4.1. Benefits for Caregivers

For physicians it can be very helpful to be informed about the symptoms and activity levels of their patients over time in real life. A smartphone/web-based application with questionnaires can be very useful in several situations and may be preferable and more patient-tailored than standardized hospital visits every few months. Monitoring in between visits can reduce the number of physician visits required, but what may be even more important is that this can be used to tailor the timing of the visits, thereby increasing the value of each visit and improving patient care [38,39,40,41]. Patients monitoring their symptoms and activity levels may improve their treatment compliance, also in sarcoidosis [35,42].

### 4.2. Benefits for Patients Themselves

Patients appreciated the use of an AT. They mentioned that it encouraged them to improve their activity levels and try to achieve personalized goals. Feedback and insights into their personal activity levels, in relation to the symptoms, was also one of the most frequently reported benefits for individual patients. The coaching part of the intervention was personalized and tailored, focusing on the personal needs of each individual participant. This was highly appreciated. Almost half of the patients without coaching (Group Ib) reported that they would have preferred additional personal coaching.

The COVID-19 pandemic has made people reluctant about visiting hospitals, for fear of becoming infected with the coronavirus. These fears have only increased as health officials and governments have advised against visiting hospitals. Hence, home monitoring and advice online or by phone has become an even more useful alternative to regular care.

### 4.3. Limitations

This study has several limitations. First, patients could not be blinded for the type of intervention (i.e., with or without coaching during the 3-months intervention period). Also, the assessor at baseline and evaluation was not blinded. In trials involving different styles of patient management, like our AT study (comparing a group receiving personal coaching with a group without coaching), full blinding is often impossible. We are aware of the risk of bias resulting from inadequate blinding, but bias associated with knowing the treatment is often subconscious. Regarding our study, one could speculate that patients in the group without coaching were less satisfied with this situation and therefore achieved less. However, this was the case only for two of the endpoints we measured (see Table 2) [43]. Secondly, although it is known that short-term results of physical training or pulmonary rehabilitation may not be sustained in the time after the training period [44], no assessment of the long-term effect of the intervention was included. Thus, it is unknown whether these benefits will sustain over a longer period of time. Nevertheless, evaluation during outpatient clinic visits revealed that most of the patients were still using the application and continued their PA. Another limitation is that the control group we used came from an earlier study by our group. However, their clinical presentation and treatment options did not differ from those of the sample used in the present study at baseline.

Patients’ daily activity levels (number of steps, estimated distance, number of flights climbed, and activity minutes) were measured and accessible to patients and their physical therapists. Unfortunately, PA data were not accessible to the researchers, and could therefore not be analyzed. It was thus not possible to report on patients’ PA levels over time.

## 5. Conclusions

Wearing an AT in general, even without personal coaching by a physical therapist, improved exercise capacity and reduced fatigue in sarcoidosis patients. Therefore, we recommend encouraging sarcoidosis patients to wear an AT, to help them improve their exercise performance and reduce fatigue. Moreover, the patients wearing an AT and receiving coaching improved their exercise capacity (SRT and SRT VO_2_max mL/kg/min), in contrast to those not receiving this coaching. Results from this study suggest that the use of wearable technology presents an opportunity to facilitate a more active lifestyle among sarcoidosis patients. Whether or not such wearable technology-based interventions and additional coaching can create sustainable behavioral changes should be the subject of future research, which should include a cost-effectiveness analysis.

## Figures and Tables

**Table 1:** Summary of demographic and clinical features of the sarcoidosis outpatient sample of the ILD Center of Excellence: Group I wearing an activity tracker (Ia with coaching; Ib without coaching), Group II: controls, no training or coaching and not wearing an activity tracker.

	Group I	Group Ia	Group Ib	Exclusion	Group II
Demographics					
Subjects, *n*	54	27	27	9	41
sex, male/female, *n*	28/26	14/13	14/13	4/5	10/31
age, years, median (range)	48 (26–72)	48 (26–65)	47 (29–72)	47 (30–69)	48 (29–73)
time since diagnosis, years, median (range)	5.0 (0–22)	4.0 (0–14)	8.0 (1–22)	9.0 (4–25)	4.0 (0–23)
BMI, kg/m^2^	27.4 ± 5.5	27.1 ± 3.6	27.8 ± 6.9	24.1 ± 4.5	27.9 ± 5.3
Treatment					
no treatment, *n*	18 (33.3%)	8 (29.6%)	10 (37.0%)	4 (44.4%)	13 (31.7%)
glucocorticoids, *n*	20 (37.0%)	11 (40.7%)	10 (37.0%)	3 (33.3%)	15 (36.6%)
Other ^#^, *n*	16 (29.6%)	8 (29.6%)	7 (26.0%)	2 (22.2%)	13 (31.7%)
Lung Function Tests					
DLCO, % predicted	77.2 ± 14.4	79.6 ± 11.8	74.7 ± 16.5	74.9 ± 20.2	77.9 ± 18.9
FEV_1_, % predicted	90.9 ± 20.9	91.0 ± 19.0	90.8 ± 22.9	66.9 ± 28.7	85.7 ± 21.8
FVC, % predicted	101.2 ± 19.4	99.9 ± 16.3	102.5 ± 22.3	97.4 ± 13.0	94.8 ± 18.0
Chest Radiographs Stages					
0/I/II/III/IV	11/12/24/4/3	6/6/12/2/1	5/6/12/2/2	0/0/2/3/4	2/10/21/3/5
Inflammatory Status					
CRP (mg/L)	4.1 ± 5.7	3.3 ± 3.1	4.9 ± 7.5	7.1 ± 14.6	4.6 ± 4.0
sIL-2R (U/mL)	4385 ± 4454	4046 ± 4710	4725 ± 4246	3008 ± 1381	6467 ± 11,807
Fatigue					
FAS	32.9 ± 7.5	33.3 ± 7.4	32.6 ± 7.6	27.8 ± 7.8	30.2 ± 9.0

Data are expressed as absolute numbers (*n*) or mean ± SD; *n* = number or median with range if appropriate; BMI: body mass index; DLCO: diffusing capacity of the lung for carbon monoxide; % predicted: percentage of predicted; FVC: forced vital capacity; FEV_1_: forced expiratory volume in 1 s; CRP: C-reactive protein; sIL-2R: soluble interleukin-2 receptor; FAS: Fatigue Assessment Scale; ^#^ immunosuppressive treatment: methotrexate with or without glucocorticoids.

**Table 2:** Summary of physical measurements of the sarcoidosis outpatient sample: Group I using an activity tracker (Ia with coaching; Ib without coaching); Group II: controls, no training.

	Group I	Group Ia	Group Ib	Group II
Subjects, number	54	27	27	41
Sex, male/female, *n*	28/26	13/14	13/14	10/31
6MWD, meters				
At baseline	566 ± 124	564 ± 138	569 ± 110	530 ± 104
At follow-up	595 ± 130	593 ± 149	597 ± 109	534 ± 110
*p*-value within groups	0.003	0.011	0.082	0.892
∆6MWD, meters	29.5 ± 69.1 *	28.7 ± 55.8	30.2 ± 81.4	4.7 ± 33.7
6MWD, % predicted				
At baseline	81.6 ± 18.1	81.4 ± 20.8	81.7 ± 15.5	75.3 ± 14.3
At follow-up	86.1 ± 17.1	85.9 ± 20.0	86.2 ± 13.8	76.1 ± 15.2
*p*-value within groups	0.001	0.001	0.049	0.832
∆ 6MWD, % predicted	4.4 ± 9.1 *	4.4 ± 6.6	4.4 ± 11.2	0.7 ± 5.0
SRT, watts				
At baseline	265 ± 79	261 ± 85.9	269 ± 74	286 ± 74
At follow-up	278 ± 85	281 ± 91.9	275 ± 80	298 ± 78
*p*-value within groups	0.004	0.005	0.269	0.497
∆ SRT, watts	12.8 ± 30.9	20.2 ± 33.8 ^#^	5.7 ± 26.4	11.6 ± 29.5
SRT, VO_2_ max, % predicted				
At baseline	25.2 ± 6.1	24.7 ± 6.6	25.7 ± 5.7	26.2 ± 6.4
At follow-up	26.4 ± 6.4	26.3 ± 6.8	26.5 ± 6.1	26.6 ± 6.7
*p*-value within groups	0.001	0.005	0.088	0.770
∆ SRT, VO_2_ max, % predicted	1.2 ± 2.5 *	1.6 ± 2.6 ^#^	0.7 ± 2.3	0.6 ± 2.4
FAS				
At baseline	32.9 ± 7.5	33.3 ± 7.4	32.6 ± 7.6	30.3 ± 9.0
At follow-up	29.1 ± 8.7	29.5 ± 8.8	28.6 ± 8.7	28.6 ± 9.0
*p*-value within groups	0.001	<0.001	0.007	0.408
∆ FAS	−3.9 ± 5.7 *	−3.7 ± 4.0	−4.0 ± 7.1	−1.8 ± 5.3

Data are expressed as absolute numbers or mean ± SD; % predicted: percentage of predicted; 6MWD: 6-minute walking distance; SRT: Steep Ramp Test; VO_2_ max: maximal oxygen uptake; FAS: Fatigue Assessment Scale. * *p* < 0.01: Group I vs. Group II; ^#^ *p* < 0.04: Group Ia vs. Group Ib).

**Table 3:** Feedback from self-report questionnaires among activity tracker users (Group I, *n* = 52).

Did You Find the Intervention Study Helpful?	
*yes*	90%
I have gained more insight into what I can do, and what I do in one day	42%
I have gained more insight into how I can best alternate activity and rest	10%
It has encouraged me to be more active	38%
*no*	10%
I already had sufficient insight	2%
It didn’t help me	6%
Monitoring my activities stimulated me too much	2%

## Data Availability

The datasets used and/or analyzed during the current study are available from the corresponding author on reasonable request. A video impression of this study can be found at: https://vimeo.com/225475968.
